# A prognostic nomogram that includes MPV in esophageal squamous cell carcinoma

**DOI:** 10.1002/cam4.6551

**Published:** 2023-10-09

**Authors:** Qiao He, Zhenglian Luo, Haiming Zou, Bo Ye, Lichun Wu, Yao Deng, Mu Yang, Dongsheng Wang, Qifeng Wang, Kaijiong Zhang

**Affiliations:** ^1^ Department of Clinical Laboratory Sichuan Clinical Research Center for Cancer, Sichuan Cancer Hospital & Institute, Sichuan Cancer Center, Affiliated Cancer Hospital of University of Electronic Science and Technology of China Chengdu China; ^2^ Department of Transfusion Medicine, West China Hospital Sichuan University Chengdu China; ^3^ Centre for Translational Research in Cancer Sichuan Clinical Research Center for Cancer, Sichuan Cancer Hospital & Institute, Sichuan Cancer Center, Affiliated Cancer Hospital of University of Electronic Science and Technology of China Chengdu China; ^4^ Department of Radiation Oncology Sichuan Clinical Research Center for Cancer, Sichuan Cancer Hospital & Institute, Sichuan Cancer Center, Affiliated Cancer Hospital of University of Electronic Science and Technology of China Chengdu China

**Keywords:** clinicopathological indicators, ESCC, MPV, OS, prognostic nomogram

## Abstract

**Background:**

Mean platelet volume (MPV), as a marker of platelet activity, has been shown to be an efficient prognostic biomarker in several types of cancer. Using MPV, this study aimed to create and validate a prognostic nomogram to the overall survival in esophageal squamous cell carcinoma (ESCC) patients.

**Methods:**

The nomogram was constructed and tested using data from a retrospective study of 1893 patients who were randomly assigned to the training and testing cohorts with a 7:3 randomization. In order to screen out the optimal predictors for overall survival (OS), we conducted the LASSO‐cox regression, univariate, and multivariate cox regression analyses. Subsequently, the predictive accuracy of the nomogram was validated in both the training and the testing cohorts. Finally, decision curve analysis (DCA) was used to confirm clinical validity.

**Results:**

Age, MPV, nerve invasion, T stage, and N stage were found as independent prognostic variables for OS and were further developed into a nomogram. The nomogram's prediction accuracy for 1‐, 3‐, and 5‐year OS was 0.736, 0.749, 0.774, and 0.724, 0.719, 0.704 in the training and testing cohorts, respectively. Furthermore, DCA results indicated that nomograms outperformed the AJCC 8th and conventional T, N staging systems in both the training and testing cohorts.

**Conclusions:**

The nomogram, in conjunction with MPV and standard clinicopathological markers, could improve the accuracy of prediction of OS in ESCC patients.

## 1 INTRODUCTION

According to the 2018 worldwide cancer data, esophageal cancer is reported to have the 6th highest cancer‐related mortality in the world.^1^ Squamous cell carcinoma was detected in roughly 90% of esophageal cancer patients in China.^2^ Most patients are diagnosed at locally advanced stages with poor prognosis due to unusual clinical signs at an early stage.

The 5‐year survival rate for esophageal cancer was only 15.3% in the 2000s.^3^ With the introduction of neoadjuvant chemoradiotherapy and developments in surgical techniques, the 5‐year survival rate in esophageal squamous cell carcinoma (ESCC) patients treated with neoadjuvant chemoradiotherapy plus surgery has grown to 59.9%.^4^ Effective clinical treatment is critical for improving ESCC survival. In clinical practice, the AJCC‐TNM staging system is the primary predictor of OS. Many studies; however, have recommended that, in addition to the AJCC staging system, additional individual characteristics should be considered in order to improve the reliability of prognostic assessments.^5^, ^6^, ^7^ The predictability and practicability of the parameters can impact their selection. For example, the unfavorable presurgery circulating tumor cell (CTC) status was independent prognostic and predictive for neoadjuvant treatment efficacy (HR = 3.652, *p* = 0.035). High epithelial CTC level (*n* ≥ 2) at the end of neoadjuvant chemoradiation therapy significantly associated with non‐pathological complete response (*p* = 0.032), shorter PFS (*p* = 3.0 × 10–6), and OS (*p* = 0.019) for locally advanced ESCC.^8^ Despite its efficacy, its clinical application is limited due to its expensive cost. Clinical laboratory hematological detection, on the other hand, is inexpensive, and widely available. Thus, predictive laboratory biomarkers such as platelet‐related measures, neutrophil‐to‐lymphocyte ratio, and C‐reactive protein/albumin ratio should be given careful consideration.

Platelets are widely established to have a crucial role in cancer progression, metastasis, and angiogenesis in a variety of ways.^8^, ^9^ Platelets may shield tumor cells from immune‐evasion by NK cells and chemokines. Liu et al.^10^ recently discovered that tumor‐educated platelets have higher migratory and cell skeleton gene expression, allowing them to travel faster and longer.

Furthermore, platelets educate tumor cells, which then efficiently transfer lipids, proteins, and RNA.^11^ The interaction between triple‐negative breast cancer cells and PLT upregulated by hypoxia is critical for malignant development.^12^ Furthermore, multiple clinical investigations have found that platelet‐related metrics such platelet count, platelet distribution width (PDW), and mean platelet volume (MPV) are widely available and efficient predictive indicators for many malignancies.^13^, ^14^, ^15^, ^16^, ^17^ Most earlier studies focused on the platelet‐to‐lymphocyte ratio (PLR), however there have been few investigations on the relationship between MPV and prognosis in ESCC. According to Ishibashi et al.,^18^ the connection between MPV and OS of ESCC remains under‐reported till the 2020s. Reduced MPV was linked to poorer survival results in esophageal cancer, according to a study published in 2018.^19^ Then, 3 years later, our research team created a useful predictive coagulation index that incorporated platelet count, MPV, and fibrinogen. This indicator has been shown to be an accurate predictor of survival in ESCC patients.^20^

In the context of this research, we hoped to create and test a novel prognostic nomogram that incorporates MPV analysis and clinical parameters to predict prognosis and support doctors in providing appropriate treatment for ESCC patients.

## 2 METHODS

### 2.1 Patients

This study included patients from Sichuan Cancer Hospital from January 2012 to December 2016. The patients were examined and chosen retrospectively using the following eligibility criteria: (1) They had pathologically proven ESCC with the resected material; (2) they did not receive any preoperative radiotherapy, chemotherapy, chemoradiotherapy, or other treatment; (3) they had presurgery hematologic detection data and clinical information. Meanwhile, we established the exclusion criteria: (1) patients had a history of cancer or any other major chronic disease; (2) the tumor penetrated the neck. With the 7:3 randomization, patients included in the trial were randomly divided into training and testing cohorts. Furthermore, we use multi‐factor stratification to increase the generalization property of both the training and testing cohorts. All of these patients were pathologically staged using the 8th edition TNM classification system of the American Joint Committee on Cancer (AJCC). This study was approved by the ethics committee of Sichuan Cancer Hospital.

### 2.2 Clinical data collection

Clinical data for the included patients were obtained from the electronic medical record system of Sichuan cancer hospital. Demographic information (age and gender), clinical data (KPS score, tumor grade, tumor site, vascular invasion, nerve invasion, dissected LN number, T stage, N stage, clinical stage, and treatment), and preoperative laboratory results (PLT (10^9^/L), MPV (FL), PDW (%), PCT (%), PT (s), INR, APTT (s), FIB (g/L), TT (s), DD (μg/mL), and FDPs (μg/mL)) were included. The clinical laboratory department of Sichuan Cancer Hospital was used to obtain the reference values for laboratory parameters.

### 2.3 Treatment and follow‐up

All patients had surgery. Patients who have one or more of the following indications for postoperative adjuvant therapy: (1) Patients with R1 or R2 resections who did not receive prior radiation. (2) Adenocarcinoma patients without preoperative radiation who had positive R0 resected lymph nodes or negative pT2‐4a lymph nodes. (3) Postoperative adjuvant can be recommended for patients with squamous carcinoma who have positive R0 resected lymph nodes or negative pT2‐4a lymph nodes and have not undergone preoperative irradiation. Radiotherapy was provided using the intensity‐modulated radiation therapy technique. Telephone calls, clinical visits, outpatient records, or messages were used to acquire regular follow‐up data. The key outcomes were 1‐, 3‐, and 5‐year overall survival (OS). OS was measured from the date of surgery to the date of death or the final follow‐up.

### 2.4 Construction and validation of the nomogram

To begin, the least absolute shrinkage and selection operator (LASSO) regression was utilized to discover the OS predictive variables, and 15 predictors conforming to the minimum lambda. Second, a univariate and multivariable cox regression analysis with 15 predictors was done to estimate the hazard ratio (HR) and 95% confidence interval of OS. Finally, the nomogram was constructed with five predictors (age, MPV, nerve invasion, T stage, and N stage) with *p* < 0.05 in multivariable cox regression. To get access to clinical use, the time receiver operating characteristic (ROC) curve, calibration curve, and decision curve analyses (DCA) were used.

### 2.5 Statistical analysis

For categorical variables, the features were expressed as number (%) and for continuous variables, median (interquartile range [IQR]). The Chi‐squared test was used to compare categorical variables. The *t*‐test was used to compare two groups of continuous variables with normal distribution. The Mann–Whitney test was performed to compare variables that did not have a normal distribution. The Bonferroni method was used to correct multiple tests. The Kaplan–Meier method was used to calculate OS probabilities. To evaluate the relationship between clinical variables and OS, cox proportional hazard models were utilized, with extra adjustments for possible confounders. In the univariate analysis, variables with a *p* < 0.05 were used to do multivariable cox regression. The optimal cut‐point for MPV was determined by using “maxstat” package. C index and Brier test values were calculated in training and test cohorts. All statistical analyses were carried out with R software 4.1.3, and a *p‐*value (two‐sided) of <0.05 was considered to indicate statistical significance.

## 3 RESULTS

### 3.1 Clinical characteristics and outcomes

The characteristics of 1327 ESCC patients in the training cohort and 566 patients in the testing cohort are described in Table 1. The median ages for the testing and the training cohorts were 62.0 (range, 57.0–67.0 years) and 63.0 (range, 57.5–68.0 years) years, respectively. The majority of patients (1559/1893, 82.4%) were males. There are no statistically significant differences between the training and the testing cohorts in clinical characteristics or laboratory tests (*p* > 0.05). Average follow‐up was 31.487 months.

**TABLE 1:** Summary descriptives of characteristic groups.

Characteristic	All (*N* = 1893)	Training (*N* = 1327)	Testing (*N* = 566)	*p*‐value
Age	62.0 [57.0, 68.0]	63.0 [57.5, 68.0]	62.0 [57.0, 67.0]	0.455
Sex				0.632
Male	1559 (82.4%)	1097 (82.7%)	462 (81.6%)	
Female	334 (17.6%)	230 (17.3%)	104 (18.4%)	
KPS_score				0.593
90–100	1071 (56.6%)	745 (56.1%)	326 (57.6%)	
70–80	822 (43.4%)	582 (43.9%)	240 (42.4%)	
Tumor_grade				0.722
Well_differentiated	399 (21.1%)	286 (21.6%)	113 (20.0%)	
Moderate_differentiation	752 (39.7%)	522 (39.3%)	230 (40.6%)	
Poorly_differentiated	742 (39.2%)	519 (39.1%)	223 (39.4%)	
Tumor_location				0.666
Lower chest	433 (22.9%)	311 (23.4%)	122 (21.6%)	
Middle chest	1039 (54.9%)	722 (54.4%)	317 (56.0%)	
Upper chest	421 (22.2%)	294 (22.2%)	127 (22.4%)	
Vascular invasion				0.365
No	1537 (81.2%)	1085 (81.8%)	452 (79.9%)	
Yes	356 (18.8%)	242 (18.2%)	114 (20.1%)	
Nerve_invasion				0.190
No	1489 (78.7%)	1055 (79.5%)	434 (76.7%)	
Yes	404 (21.3%)	272 (20.5%)	132 (23.3%)	
Dissected LN number	20.0 [14.0, 28.0]	21.0 [15.0,28.0]	20.0 [14.0,28.0]	0.273
pT stage				0.511
T1	228 (12.0%)	161 (12.1%)	67 (11.8%)	
T2	390 (20.6%)	271 (20.4%)	119 (21.0%)	
T3	1111 (58.7%)	788 (59.4%)	323 (57.1%)	
T4	164 (8.7%)	107 (8.1%)	57 (10.1%)	
Treatment				0.792
Surgery	1054 (55.7%)	742 (55.9%)	312 (55.1%)	
Surgery + RT	37 (1.9%)	28 (2.1%)	9 (1.6%)	
Surgery + CT	609 (32.2%)	420 (31.7%)	189 (33.4%)	
Surgery + CCRT	193 (10.2%)	137 (10.3%)	56 (9.9%)	
Statue				0.590
Alive	1103 (58.3%)	779 (58.7%)	324 (57.2%)	
Dead	790 (41.7%)	548 (41.3%)	242 (42.8%)	
pN stage				0.940
N0	845 (44.6%)	588 (44.3%)	257 (45.4%)	
N1	567 (30.0%)	403 (30.4%)	164 (29.0%)	
N2	336 (17.7%)	234 (17.6%)	102 (18.0%)	
N3	145 (7.7%)	102 (7.7%)	43 (7.6%)	
pAJCC8th stage				1.000
I	231 (12.2%)	162 (12.2%)	69 (12.2%)	
II	603 (31.9%)	423 (31.9%)	180 (31.8%)	
III	854 (45.1%)	598 (45.1%)	256 (45.2%)	
IV	205 (10.8%)	144 (10.9%)	61 (10.8%)	
PLT	175 [138, 219]	174 [137, 218]	182 [141, 223]	0.160
MPV	11.5 [10.3, 12.7]	11.5 [10.3, 12.6]	11.6 [10.4, 12.8]	0.441
PDW	16.3 [16.1, 16.6]	16.3 [16.1, 16.6]	16.3 [16.1, 16.6]	0.650
PCT	0.2 [0.17, 0.24]	0.2 [0.17, 0.24]	0.2 [0.17, 0.25]	0.056
PT	16.6 [15.4, 17.7]	16.7 [15.5, 17.8]	16.5 [15.3, 17.7]	0.103
INR	0.9 [0.88, 0.98]	0.9 [0.89, 0.98]	0.9 [0.88, 0.98]	0.970
APTT	30.5 [27.3, 34.9]	30.6 [27.4, 34.9]	30.4 [27.2, 34.8]	0.706
FIB	3.3 [2.7, 3.8]	3.3 [2.7, 3.8]	3.3 [2.8, 3.9]	0.498
TT	16.8 [15.8, 17.9]	16.9 [15.8, 18.0]	16.6 [15.6, 17.8]	0.053
DD	0.3 [0.2, 0.4]	0.3 [0.2, 0.4]	0.3 [0.2, 0.4]	0.095
FDPs	1.4 [1.1, 1.8]	1.4 [1.1,1.8]	1.4 [1.1, 1.8]	0.209

Abbreviations: APTT, prothrombin time; CCRT, concurrent chemoradiotherapy; CT, chemotherapy; DD, D‐Dimer; FDPs, fibrinogen degradation products; FIB, fibrinogen; INR, International normalized ratio; KPS, Karnofsky Performance Scale; MPV, mean platelet volume; PCT, platelet hematocrit; PDW, platelet distribution width; PLT, platelet count; pN, pathological N; pT, pathological T; PT, prothrombin time; RT, radiotherapy; TT, fibrinogen.

### 3.2 Nomogram development in the training cohort

LASSO‐logistic regression was used to pick out the best predictors of OS from the 24 parameters provided in Table 1.

**FIGURE 1:**
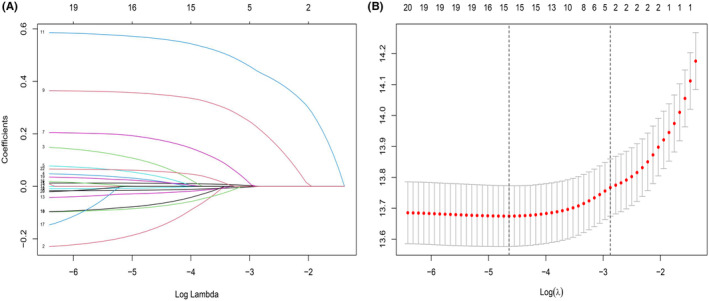
Selection of risk factors for OS with least absolute shrinkage and selection operator (LASSO) cox regression model. (A) LASSO Cox regression for the 15 risk factors. (B) Cross‐validation for the LASSO Cox regression.

**FIGURE 2:**
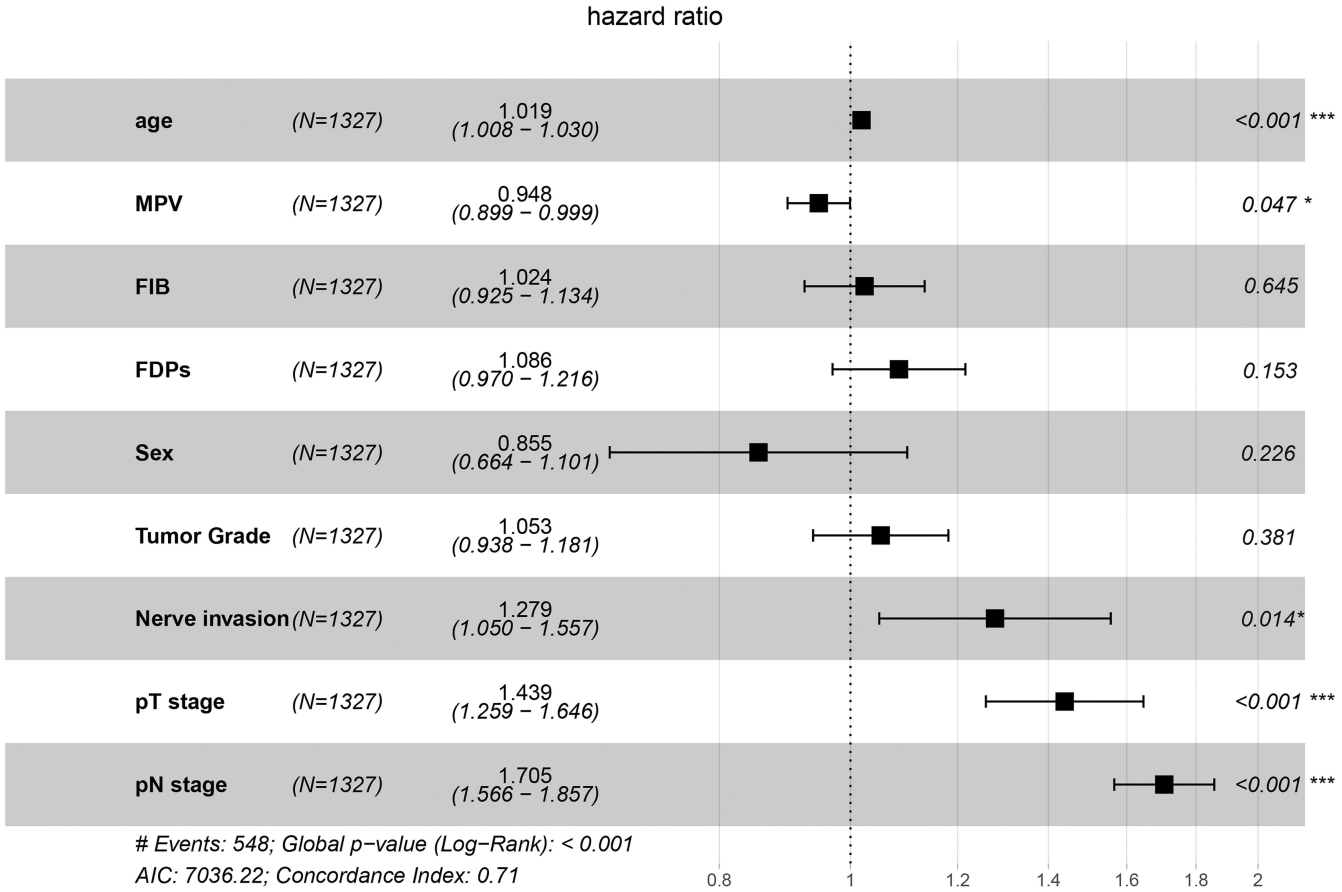
Forest plot of the multivariable Cox proportional hazards regression analyses was conducted with 9 risk factors, which had *p* < 0.05 in univariate analysis. FIB, fibrinogen; FDPs, fibrinogen degradation products; MPV, mean platelet volume.

**FIGURE 3:**
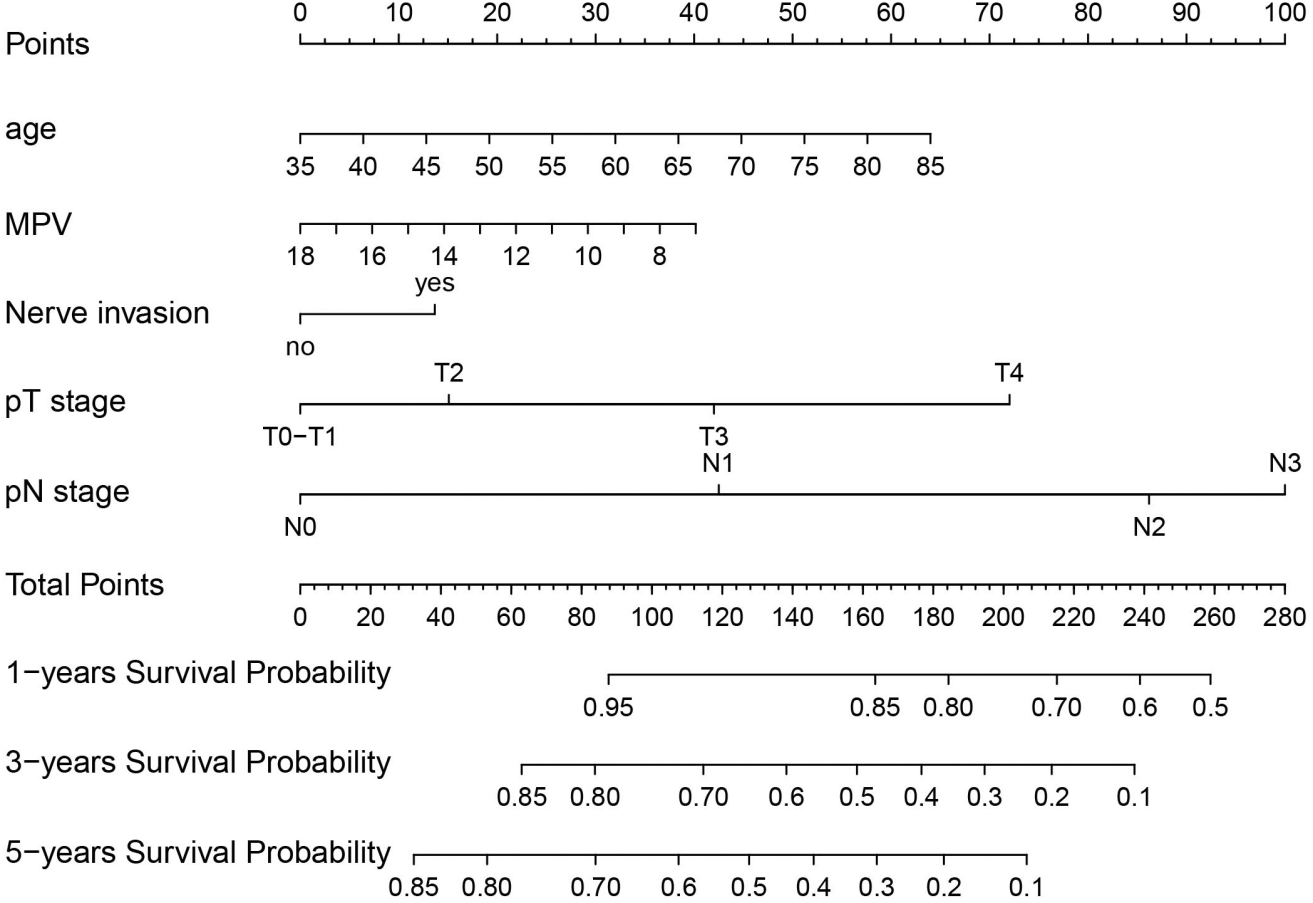
Prognostic nomogram for 1, 3, and 5 years was developed in training cohort. Total points were sum of age, MPV, nerve invasion, T stage, and N stage, MPV, mean platelet volume.

As shown in Figure 1, 15 variables with a minimum value of lambda (λ) were chosen. Subsequently, cox regression analysis was undertaken to investigate independent prognostic variables for OS. According to the univariate analysis: age, FDPs, FIB, MPV, nerve invasion, gender, T stage, N stage, and tumor Grade could be candidate variables for the multivariate analysis (*p* < 0.05, Table 2). The multivariate analysis verified that age, MPV, nerve invasion, T stage, and N stage were independent predictive variables for OS (*p* < 0.05, Table 2, Figure 2). The prognostic nomogram was created in the training cohorts based on these independent prognostic criteria (Figure 3). Furthermore, we investigated the possible prognostic value of MPV in ESCC patients. Patients with MPV ≤11.8 fL had a dismal prognosis, as shown in Figure S1. Given this, MPV could serve as an independent prognosis predictor of ESCC.

**TABLE 2:** Univariate and multivariable Cox proportional hazards regression analyses for the prediction of OS.

Variable	Univariate		Multivariable	
	HR (95% CI)	*p‐value*	HR (95% CI)	*p‐value*
Age	1.010 (1.000–1.021)	**0.040**	1.019 (1.008–1.030)	**<0.001**
APTT	0.997 (0.984–1.009)	0.595		
Dissected LN number	0.998 (0.991–1.006)	0.671		
FDPs	1.147 (1.031–1.277)	**0.012**	1.086 (0.970–1.216)	0.152
FIB	1.182 (1.075–1.299)	**0.001**	1.024 (0.925–1.134)	0.645
KPS score	1.156 (0.975–1.370)	0.096		
MPV	0.923 (0.877–0.972)	**0.002**	0.948 (0.899–0.999)	**0.047**
Nerve invasion	1.618 (1.338–1.956)	**<0.001**	1.279 (1.050–1.557)	**0.014**
PDW	0.888 (0.777–1.015)	0.082		
Sex	0.679 (0.530–0.870)	**0.002**	0.855 (0.664–1.101)	0.226
pT stage	1.775 (1.571–2.006)	**<0.001**	1.439 (1.259–1.646)	**<0.001**
pN stage	1.821 (1.682–1.972)	**<0.001**	1.705 (1.566–1.857)	**<0.001**
Treatment	0.997 (0.925–1.073)	0.925		
Tumor grade	1.217 (1.089–1.359)	**0.001**	1.053 (0.938–1.181)	0.381
Tumor location	1.044 (0.924–1.180)	0.486		

Abbreviations: APTT, prothrombin time; FDPs, fibrinogen degradation products; FIB, fibrinogen; KPS, Karnofsky Performance Scale; LN, lymph node; MPV, mean platelet volume; OS, overall survival; PDW, platelet distribution width; pN pathological N; pT, pathological T.

The bold formatting indicates statistically significant *p*‐values, highlighting that age, mean platelet volume (MPV), nerve invasion, pathological T (pT) stage, and pathological N (pN) stage were significant independent prognostic factors for overall survival in patients with esophageal squamous cell carcinoma (ESCC). Specifically, in both univariate and multivariate analyses, older age, lower MPV levels, presence of nerve invasion, higher pT stage, and positive pN stage were associated with poorer survival outcomes. These clinicopathological characteristics may serve as useful predictive markers of prognosis in ESCC.

### 3.3 Validation of the predictive accuracy of the nomograms for OS

**FIGURE 4:**
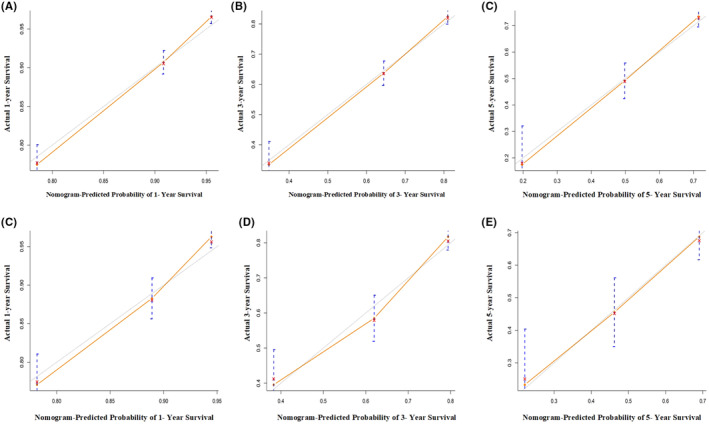
The calibration curves of the nomogram in training cohort (A‐C) and testing cohort (D‐F) for predicting 1, 3, and 5 years OS. Nomogram‐predicted probability of OS is plotted on the x‐axis; actual overall survival is plotted on the y‐axis.

Calibration curves were applied to validate the nomogram's ability to predict 1‐, 3‐, and 5‐year OS. For survival rates, calibration curves showed higher agreement between nomogram prediction and actual observations. The training cohort (Figure 4A–C) and testing cohort (Figure 4D–F) nomograms showed good consistency in OS prediction.

**FIGURE 5:**
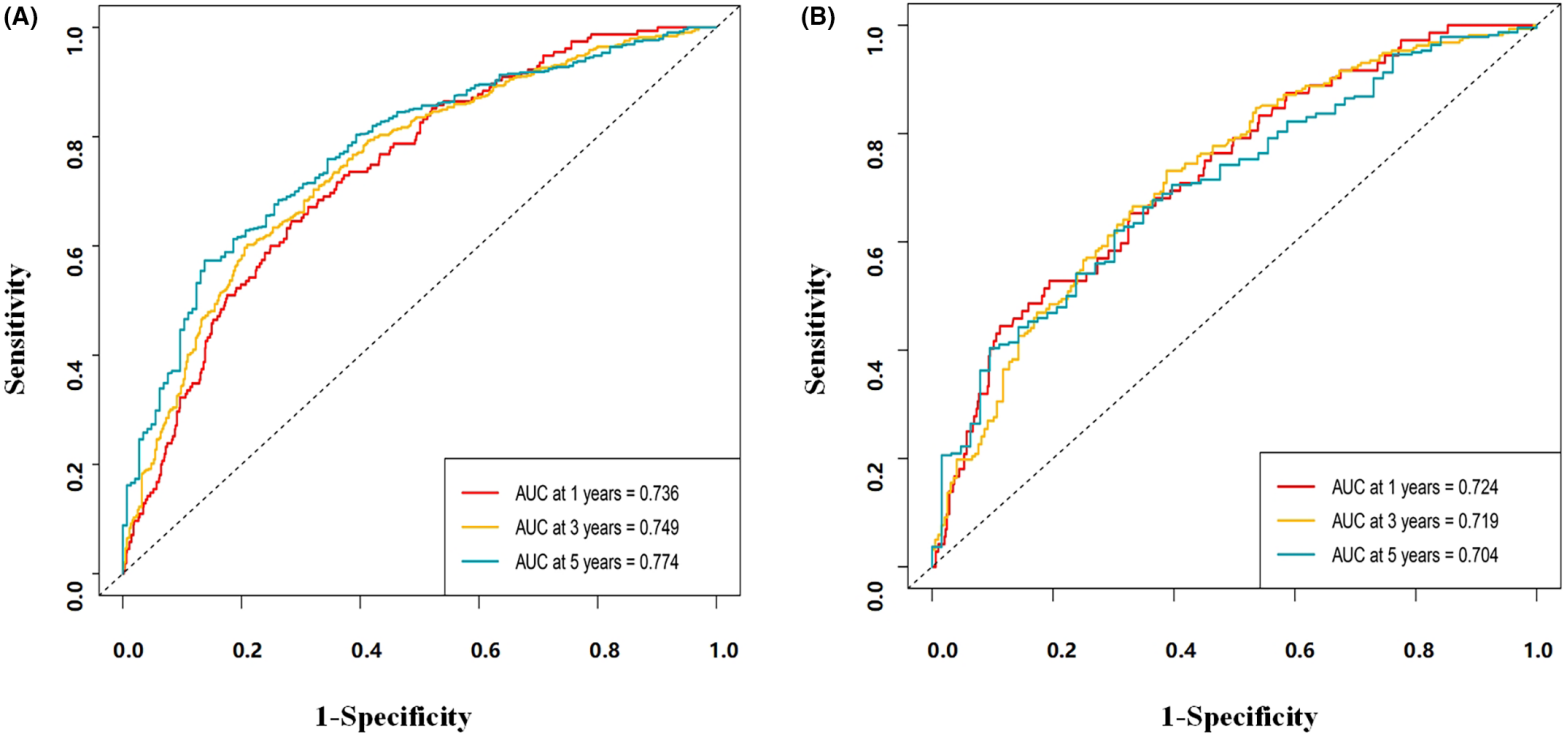
Receiver operating characteristic (ROC) curves for 1, 3, and 5 years OS based on the nomogram constructed in training cohort (A) and testing cohort (B). AUC, area under the curve.

In addition, the predicted accuracy was evaluated with time‐dependent receiver operating characteristic (time‐ROC). Figure 5 depicts the outcomes of the training and the testing cohort. In the training and the testing cohorts, the AUC for 1‐, 3‐, and 5‐year OS were 0.736, 0.749, 0.774, and 0.724, 0.719, 0.704, respectively. C index and Brier test values in training and test cohorts were 0.707, 0.163; 0.695, 0.173, respectively.

### 3.4 Comparison of predictive accuracy between nomogram and conventional stage systems

**FIGURE 6:**
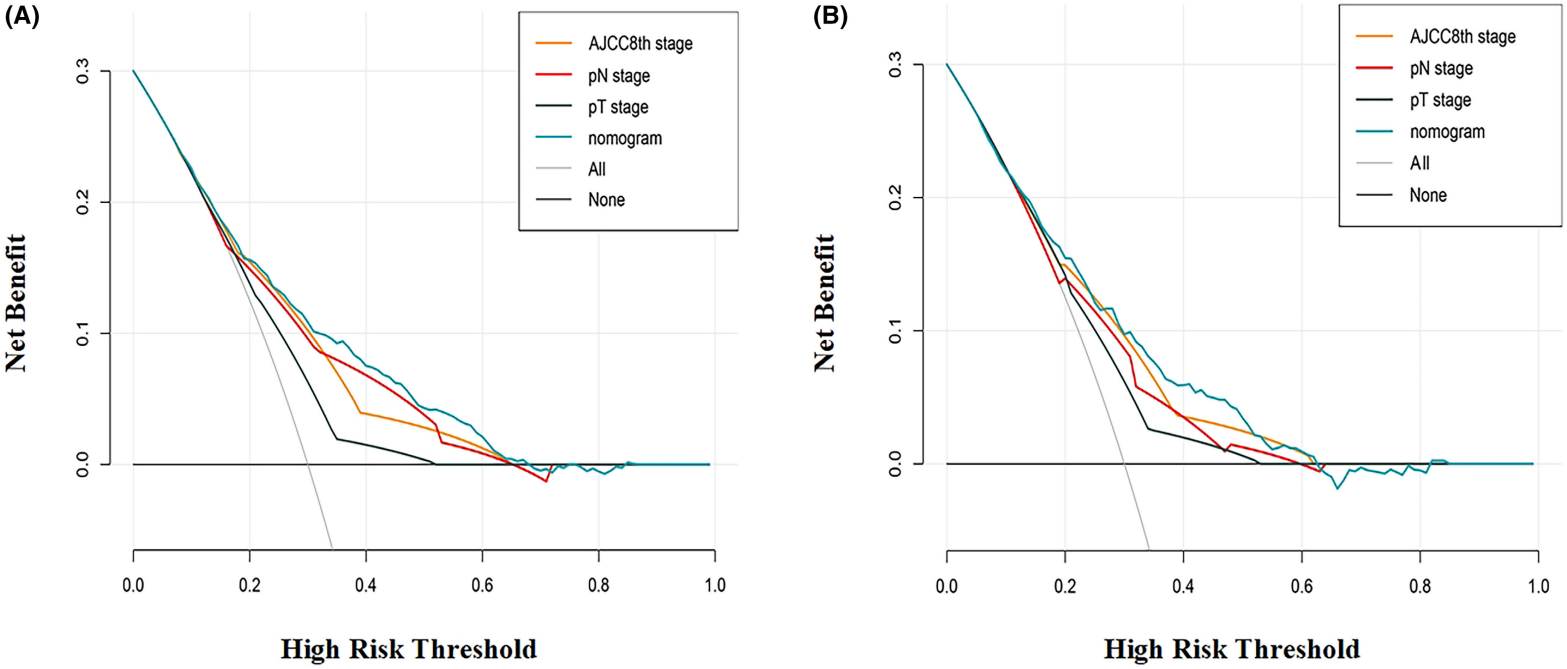
Comparison the decision curves analysis (DCA) curve between nomogram and conventional staging systems in training cohort (A) and testing cohort (B).

Finally, DCA was used to validate the nomogram's clinical validity. The nomogram's prediction power was compared to the AJCC 8th stage, T, N stage scheme. The advantage of the MPV nomogram outperformed the AJCC 8th, T, and N stages in both the training and testing cohorts (Figure 6).

## 4 DISCUSSION

As is widely documented, the initial tumor site, treatment, smoking status, histology, older age, TNM stage, and vascular invasion all have a significant impact on the OS of ECSS patients.^21^, ^22^ In addition to these traditional clinicopathological indications, additional, and individualized predictors have recently been developed. Clinical laboratory hematological markers (pre‐PLT, post‐Hb, and △WBC),^23^ inflammatory score ADS (Alb to fibrinogen ratio‐Alb‐derived neutrophil/lymphocyte ratio score),^24^ platelet‐related parameters, serum apolipoprotein A‐I,^25^ coagulation Index,^20^ and autophagy‐related three‐gene PARP1, ITGA6, and FADD)^26^ have been shown to accurately predict OS for ESCC. Currently, nomograms are mostly utilized to predict endoscopic screening, treatment efficacy, lymph node metastasis, and OS for ESCC.^7^, ^27^, ^28^, ^29^ To the best of our knowledge, this is the first study to construct and evaluate an ESCC prognostic nomogram in conjunction with MPV and standard clinicopathological markers. The nomogram performed predicted 1‐, 3‐, and 5‐year OS well, which was supported by calibration curves and decision curve analysis.

In clinical practice, MPV is commonly referred to the mean size of platelets. Many studies on new perspectives for MPV in inflammation and carcinoma have been conducted over the last few decades. MPV has been identified as a useful prognostic biomarker in cancer and cardiovascular disorders.^30^ Low In renal cell carcinoma, MPV has been linked to decreased cancer‐specific survival and a higher risk of recurrence.^31^ Oropharyngeal cancer and oral squamous cell carcinoma (Stage III and IV) had unfavorable prognoses when pretreatment MPV was decreased.^32^, ^33^ Furthermore, Li et al. and Shen et al.^19^, ^34^ discovered that lower MPV predicted poorer survival outcomes in breast cancer patients with type 2 diabetes and esophageal cancer. However, in a study of 509 colorectal cancer patients, higher MPV was associated with a poor OS (*p* = 0.035).^35^ The findings for pancreatic cancer patients with synchronous liver metastases were supported by this finding.^36^ To summarize, the prognosis value of MPV may vary greatly in different types of cancer with different characteristics of oncogenesis. As evidenced in colorectal cancer, colorectal cancer cells secreted IL‐6, and stimulated platelet production by increasing thrombopoietin secretion.^37^ In line with the previous studies, our findings show that MPV can be considered as an independent prognostic factor for OS in ESCC (*p* < 0.05).

The underlying mechanisms and signaling have not been thoroughly studied. In a previous study, Shen et al.^19^ explained in a previous study that decreased MPV indicated increased consumption of large platelets in inflammatory states with high‐grade inflammation. Zhang et al.^38^ conducted the first COP–MPV (combination of preoperative PLT and MPV) study in 2016 to find a significant association between OS and DFS in I–III ESCC. The researchers discovered that a low MPV level was roughly equivalent to active inflammation. Patients with resectable ESCC who had a low MPV/PLT count ratio (≤0.04) had a lower cancer‐specific survival.^39^ MPV and PLT count are two important variables to consider when assessing platelet activation. Increased pro‐inflammatory cytokines IL‐6, IL‐1, and TNF‐*α* contribute to platelet increase in ESCC.^40^ Larger platelets are then consumed in the inflammatory microenvironment. Furthermore, platelet‐derived regulatory factors such as EGF and PDGF promote angiogenesis in advanced ESCC.^41^ These findings suggest that MPV plays an important role in predicting ESCC survival.

MPV, which had never been studied in ESCC patient nomograms, was combined with age, nerve invasion, T and N stage in our study to create an effective survival prognosis nomogram. The accuracy and efficacy of OS prediction for ESCC were evaluated with ROC. The AUC of the nomograms was more than 0.7 in both the training and the testing cohorts. Furthermore, when compared to the T, N staging system, the nomogram proposed in the study was found to be more accurate and superior. Despite the benefits, there are some drawbacks that should not be overlooked. First, because this was a retrospective study, the nomogram was only tested in one study center. As a result, a multicenter confirmation is required to confirm its efficacy. Second, because our study's endpoint was OS, the prediction value for progression‐free survival or disease‐free survival was not included. In addition to tumors, platelets are influenced by infection, medication, and other variables. These shortcomings serve as an invitation for future research in this area.

## 5 CONCLUSIONS

This is the first time to construct and evaluate an ESCC prognostic nomogram in conjunction with MPV and standard clinicopathological markers. The finding of this study indicated the proposed nomogram accurately predicted the OS for ESCC.

## AUTHOR CONTRIBUTIONS

**Qiao He:** Writing – original draft (equal). **zhenglian luo:** Writing – original draft (equal). **Haiming Zou:** Conceptualization (equal); data curation (equal). **bo ye:** Data curation (equal); investigation (equal). **lichun wu:** Investigation (equal); methodology (equal). **Yao Deng:** Investigation (equal); methodology (equal). **Mu Yang:** Conceptualization (equal); project administration (equal). **Dongsheng Wang:** Conceptualization (equal); funding acquisition (equal); supervision (equal). **qifeng wang:** Conceptualization (equal). **kaijiong Zhang:** Conceptualization (equal); methodology (equal); project administration (equal); validation (equal).

## CONFLICT OF INTEREST STATEMENT

The authors state that there are no conflicts of financial interest or otherwise regarding the publication of this article.

## ETHICS STATEMENT

The study was approved by the ethics committee of the Institutional Ethics Committee of Sichuan Cancer Hospital (SCCHEC‐02‐2020‐015) and carried out in accordance with relevant guidelines and regulations. All patients included in this study signed informed consent statements.

## Supporting information


Figure S1.

## Data Availability

Data are available from the corresponding author upon reasonable request.
