# Supplementary figures and images for: A prognostic nomogram that includes MPV in esophageal squamous cell carcinoma
Source: Cancer Med. 2023 Oct 9;12(20):20266–76. doi: 10.1002/cam4.6551 (PMC10652314; doi:10.1002/cam4.6551)

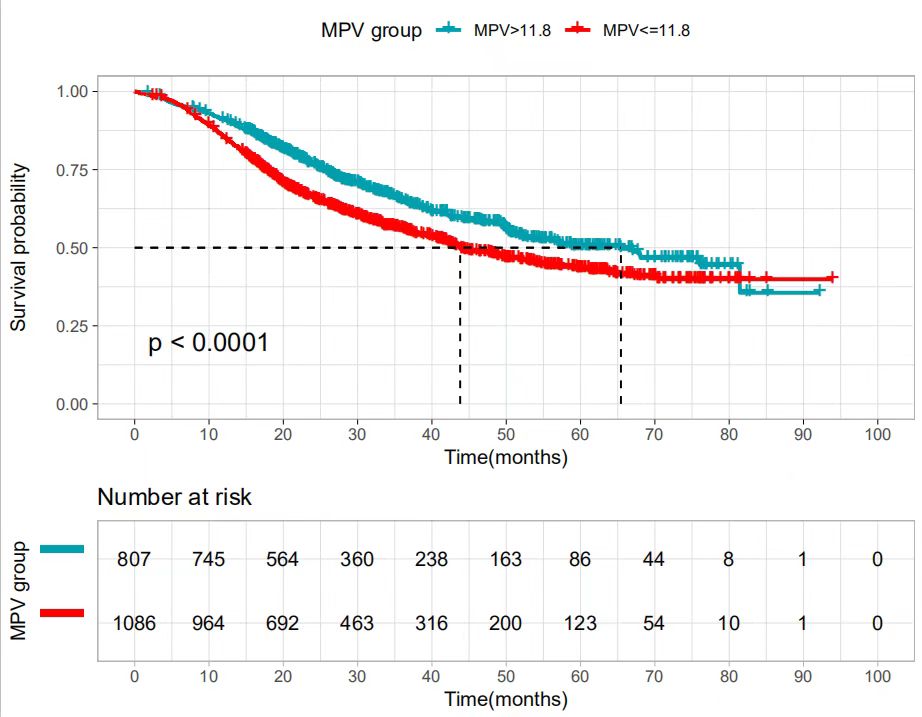

Supplement: Supplementary file 1 — Figure S1. [file CAM4-12-20266-s001.jpg]
